# Emotion Regulation and Mood during the COVID-19 Pandemic

**DOI:** 10.3390/jcm12082758

**Published:** 2023-04-07

**Authors:** Joris C. Verster, Pauline A. Hendriksen, Pantea Kiani, Agnese Merlo, Jessica Balikji, Johan Garssen, Gillian Bruce

**Affiliations:** 1 Division of Pharmacology, Utrecht Institute for Pharmaceutical Sciences, Utrecht University, 3584 CG Utrecht, The Netherlands; p.a.hendriksen@students.uu.nl (P.A.H.); p.kiani@uu.nl (P.K.); a.merlo@uu.nl (A.M.); j.balikji@uu.nl (J.B.); j.garssen@uu.nl (J.G.); 2 Centre for Human Psychopharmacology, Swinburne University, Melbourne, VIC 3122, Australia; 3 PanGenix, Elektraweg 5, 3144 CB Maassluis, The Netherlands; 4 Global Centre of Excellence Immunology, Nutricia Danone Research, 3584 CT Utrecht, The Netherlands; 5 Division of Psychology and Social Work, School of Education and Social Sciences, University of the West of Scotland, Paisley PA1 2BE, UK; gillian.bruce@uws.ac.uk

The 2019 coronavirus disease (COVID-19) pandemic has had a significant negative impact on health, mood, and well-being [1,2]. The risk of becoming infected with SARS-CoV-2 and the possible consequences of hospitalization or death fueled the fear of COVID-19, particularly among vulnerable groups at risk, such as the elderly and those with non-communicable diseases and mental health disorders [3,4]. Governments around the world installed measures with the aim of preventing the spread of the coronavirus. These measures included hygiene instructions such as washing hands, maintaining a safe distance from other individuals, wearing facemasks, frequently ventilating rooms, and staying home if infected with SARS-CoV-2 (quarantine) [5]. In addition, many countries enforced travel restrictions, and some even closed their borders [6]. Within countries, stay-at-home orders (i.e., a lockdown) were often enforced. During these lockdown periods, people were instructed to work from home. For students, the pandemic resulted in an abrupt transition from face-to-face teaching to online education [7]. Bars, restaurants, and other social venues closed their doors, and people were instructed to leave their homes only for necessary groceries or to visit a doctor or pharmacy. However, there was great variability in the strength of these measures between different countries and in the duration of lockdown periods [8]. Some countries refrained from adopting lockdown periods. While the primary purpose of lockdowns was to reduce harm by reducing the spread of the coronavirus, a recent meta-analysis revealed that the lockdown measures had a negligible impact on COVID-19 mortality (i.e., a 0.2% reduction) [9]. The latter should be considered an important lesson learned from the COVID-19 pandemic, since the lockdown measures did have significant and profound negative effect on mood, quality of life, and health [1,2].

A clear example of such mood effects is illustrated by the study by Hendriksen et al. [10,11]. In this study among Dutch students, mood was assessed retrospectively across four time spans: for the period before the COVID-19 pandemic, the first lockdown period, the subsequent period without lockdown, and a second lockdown period. The analysis revealed a significantly poorer mood during the two lockdown periods (See Figure 1), including significantly increased stress, loneliness, and depression. Similar negative mood effects were found for anxiety, fatigue, happiness, and optimism. Overall, the lockdowns were associated with a significantly reduced quality of life. However, the lockdown effects were not limited to mood effects. As is evident from Figure 1, health correlates such as sleep quality were also significantly affected during the lockdown periods [10,11]. For future pandemics, it is essential to weigh the limited advantages of lockdown periods in terms of reducing the spread of SARS-CoV-2 against their significant negative impact on mood, health, and quality of life.

Other studies revealed great variability in how people coped with mood changes (i.e., emotion regulation) during the COVID-19 pandemic [13,14]. The extent to which individuals are capable of bouncing back (i.e., mental resilience) and coping with major life events (such as the fear of COVID-19 or a lockdown period) has a direct impact on the psychological distress that accompanies such a life event. Thus, better emotion regulation has been associated with experiencing less psychological distress during the COVID-19 pandemic [13,14].

A positive, good mood is associated with good health, whereas a poor mood is associated with poor health [15,16]. This relationship is bi-directional, i.e., mood impacts health, and health status affects mood. The latter explains why changes in mood, or the absence of adequate mood regulation, can impact the susceptibility to becoming infected with SARS-CoV-2 and the number and severity of symptoms experienced when suffering from COVID-19. Using data from Hendriksen et al. [10,11], this relationship is further explained in Figure 2. Figure 2A shows the correlations between immune fitness prior to the COVID-19 pandemic (assessed for 2019 with the immune status questionnaire, ISQ [17]) and mood during the first lockdown period. It is evident that immune fitness before the COVID-19 pandemic (ISQ) is a significant predictor of mood during the first lockdown period. Figure 2B shows the correlations between mood before the COVID-19 pandemic and immune fitness (assessed with a single-item scale ranging from 0 (very poor) to 10 (excellent) [18]) during the first lockdown period. It is evident that mood before the COVID-19 pandemic is also a significant predictor of immune fitness during the first lockdown period. Figure 2C summarizes the model that describes the impact of the COVID-19 lockdown and its restrictions on mood and immune fitness, and how these may subsequently affect the number and severity of COVID-19 symptoms. The model also acknowledges the impact of emotion regulation, lifestyle, and health correlates on these relationships.

Kiani et al. [19] investigated the relationship between immune fitness before the COVID-19 pandemic as a predictor of COVID-19 symptom severity once infected with SARS-CoV-2. The analysis of data from n = 87 Dutch adults with a confirmed SARS-CoV-2 infection included many potential predictors of COVID-19 symptom presence and severity, such as demographics (e.g., sex and age), body mass index (BMI), and the presence of underlying diseases. The analysis revealed a model showing that immune fitness before the COVID-19 pandemic was the only predictor of the number (27.2%) and severity (33.1%) of COVID-19 symptoms during the pandemic [19]. Figure 3 shows the significant correlation between immune fitness before the COVID-19 pandemic and COVID-19 symptom severity during the pandemic.

Lifestyle and health status can differentially impact mood and immune fitness, the susceptibility to become infected with SARS-CoV-2, and the number and severity of symptoms experienced when suffering from COVID-19. Examples of the impact of lifestyle factors that may affect mood and immune fitness include daily diet [20], physical activity [21], BMI [22], sleep [23], and substance use [24]. In addition, social factors, such as marital status and living situation, are related to emotion regulation and coping ability. For example, a recent study demonstrated that those who lived alone during the first Dutch COVID-19 lockdown reported significantly greater negative mood changes compared to individuals that lived together with others [25].

Alcohol is an example of how lifestyle impacted mood and immune fitness during the COVID-19 pandemic. The lockdown periods often included the closure of bars, restaurants, clubs, and other venues where alcohol is usually consumed. Particularly for younger adults (e.g., students), this often resulted in a significant reduction in alcohol consumption and a reduction in the frequency and severity of alcohol hangovers [26,27,28]. Interestingly, despite the transition from face-to-face teaching to online education and the significantly limited interactions with teachers and other students [11], the reduction in alcohol consumption and hangovers was associated with improved academic performance during the COVID-19 pandemic [29]. However, a substantial subsample of other drinkers (~22%) increased their alcohol consumption during the lockdown periods [26]. For this group, increased alcohol consumption and more frequently experienced hangovers were associated with poorer mood and poorer immune fitness [24].

Finally, it is important to identify possible groups at risk. Having underlying diseases (e.g., cardiovascular disease or diabetes) is one of the risk factors for critical illness and COVID-19-related mortality [30]. A systematic review and meta-analysis of 634,338 COVID-19 patients revealed that having mental disorders was also associated with an unfavorable disease course once infected with SARS-CoV-2, including greater symptom severity and increased mortality rates [31]. A recent study demonstrated this difference by comparing individuals with and without self-reported impaired wound healing (e.g., slow-healing wounds and infections) (See Figure 4) [32]. Previous studies showed that impaired wound healing was associated with a poorer mood and quality of life [33], poorer immune fitness and experiencing more immune-related complaints [34], and various negative health consequences, such as poorer sleep quality [35] and experiencing more gastrointestinal complaints [36]. During the COVID-19 pandemic lockdown periods, the effects on mood and quality of life was significantly more profound in individuals with self-reported impaired wound healing compared to the healthy control group. In turn, poorer mood and quality of life were accompanied by a significantly poorer immune fitness (See Figure 4).

In conclusion, regulation of emotions and mood has a significant impact on immune fitness and therefore on the susceptibility to becoming infected with SARS-CoV-2 and the number and severity of symptoms experienced when suffering from COVID-19. More research is therefore warranted. In particular, research on populations at risk (e.g., elderly individuals and subjects with an underlying disease) deserves more attention. With respect to pandemic preparedness, mental resilience and emotion regulation play an essential role and have a clear, direct impact on immune fitness. Prevention should therefore focus on supporting a healthy lifestyle and enhancing adequate coping strategies to lower the susceptibility to becoming infected with future viruses and reduce the severity of symptoms experienced once infected.

## Figures and Tables

**Figure 1:**
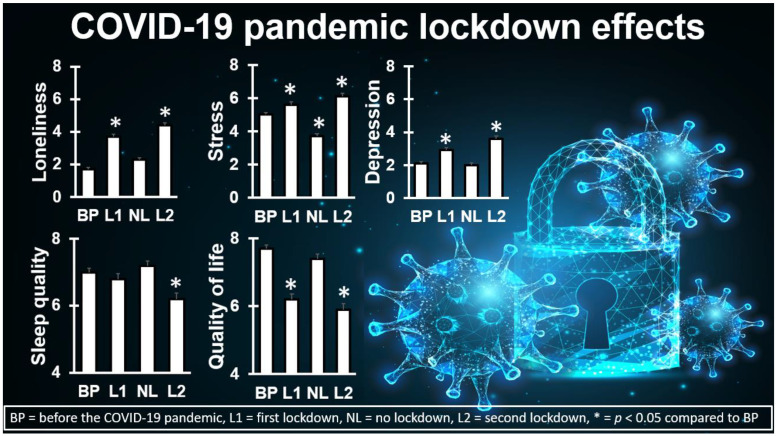
Examples of COVID-19 lockdown effects among Dutch university students. Loneliness, stress, and depression were assessed on a scale ranging from 0 (absent) to 10 (extreme) [12]. Sleep quality and quality of life were assessed on a scale ranging from 0 (very poor) to 10 (excellent) [12]. Significant (*p* < 0.0167) negative effects of lockdown were found for all measures. Data from references [10,11]. Permission to use the background image was obtained from Depositphotos.

**Figure 2:**
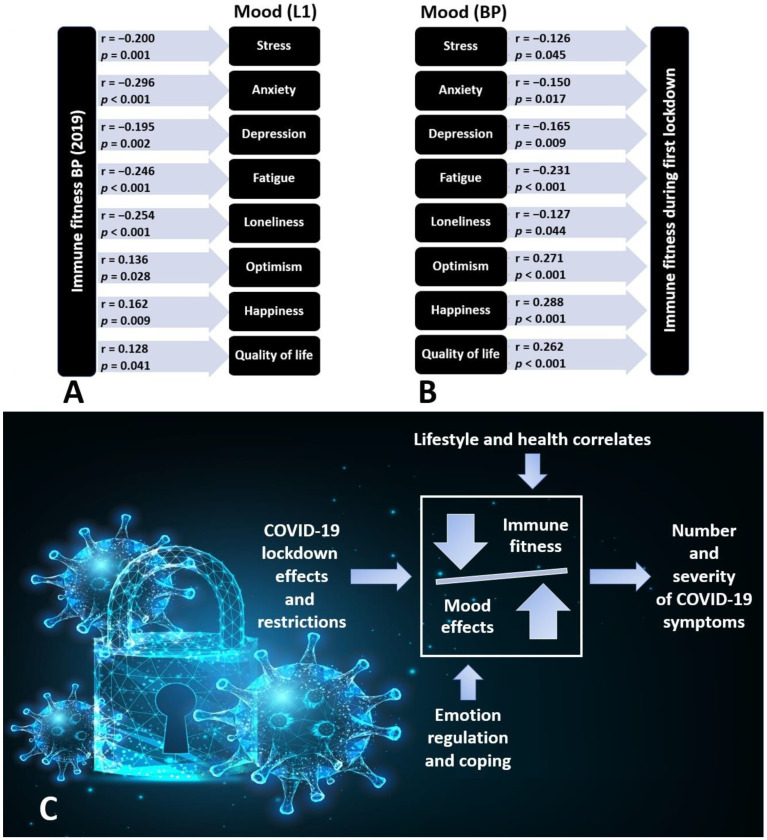
The bi-directional relationship between mood and immune fitness. Spearman’s correlations and *p*-values are shown. (**A**) Shows the correlations between immune fitness before the COVID-19 pandemic (assessed for 2019 with the ISQ [17]) and mood during the first lockdown period. (**B**) Shows the correlations between mood before the COVID-19 pandemic and immune fitness during the first lockdown period. (**C**) Summarizes the model that describes the impact of COVID-19 lockdown effects and its restrictions on mood and immune fitness, and the number and severity of COVID-19 symptoms. Abbreviations: BP = before the COVID-19 pandemic; L1 = the first lockdown period; ISQ = immune status questionnaire; COVID-19 = 2019 coronavirus disease. Immune fitness during the first lockdown period was assessed on a scale ranging from 0 (very poor) to 10 (excellent) [18]. Correlations were considered significant after Bonferroni’s correction if *p* < 0.00625. Data from references [10,11]. Permission to use the background image of (**C**) was obtained from Depositphotos.

**Figure 3:**
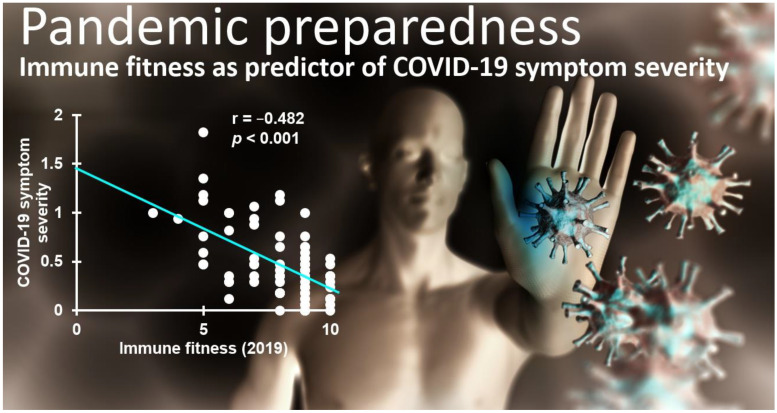
Relationship between immune fitness and the severity of COVID-19 symptoms. Immune fitness was assessed for 2019 with the Immune Status Questionnaire (ISQ) [17]. COVID-19 symptom severity was assessed in 2020–2021 in n = 87 subjects with confirmed infection with SARS-CoV-2. Data from reference [19]. Permission to use the background image was obtained from Depositphotos.

**Figure 4:**
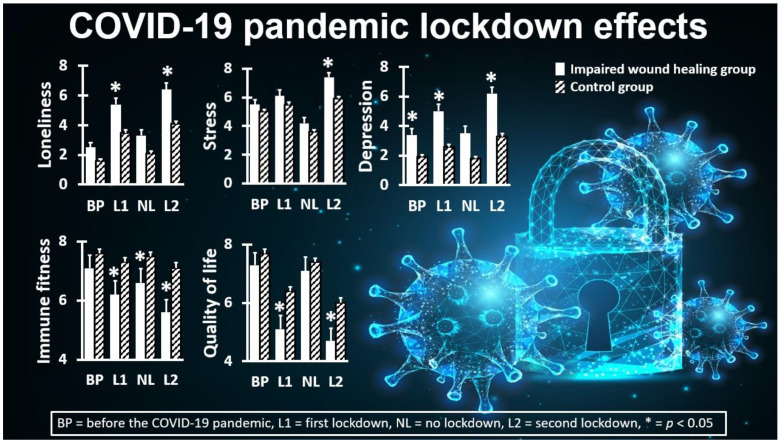
Examples of COVID-19 lockdown effects among individuals with and without self-reported impaired wound healing. Loneliness, stress, and depression were assessed on a scale ranging from 0 (absent) to 10 (extreme) [12]. Sleep quality and quality of life were assessed on a scale ranging from 0 (very poor) to 10 (excellent) [12]. For each time period, significant (*p* < 0.0167) differences between individuals with impaired wound healing (striped bars) and individuals without impaired wound healing (white bars) are indicated with *. Data from references [10,32]. Permission to use the background image was obtained from Depositphotos.
